# Supplementary material for: Different analysis strategies of 16S rRNA gene data from rodent studies generate contrasting views of gut bacterial communities associated with diet, health and obesity
Source: PeerJ. 2020 Nov 17;8:e10372. doi: 10.7717/peerj.10372 (PMC7678494; doi:10.7717/peerj.10372)
Supplement: Supplemental Information 1 [file peerj-08-10372-s001.docx]

**SUPPLEMENTAL INFORMATION**

**Different analysis strategies of 16S rRNA gene data from rodent studies generate contrasting views of gut bacterial communities associated with diet, health and obesity**

Jose F. Garcia-Mazcorro^1,*^, Jorge R. Kawas^2^, Cuauhtemoc Licona-Cassani^3^, Susanne U. Mertens-Talcott^4^, Giuliana Noratto^4^

^1^ Research and Development, MNA de Mexico, San Nicolas de los Garza, Nuevo Leon, Mexico

^2^ Faculty of Agronomy, Universidad Autonoma de Nuevo Leon, General Escobedo, Nuevo Leon, Mexico

^3^ School of Engineering and Sciences, Tecnologico de Monterrey, Monterrey, Nuevo Leon, Mexico

^4^ Department of Nutrition and Food Science, Texas A&M University, College Station, Texas, USA

Corresponding Author:

Jose Garcia-Mazcorro^1^

Avenida Acapulco 770, San Nicolas de los Garza, Nuevo Leon, 66477, Mexico

Email address: [josegarcia_mex@hotmail.com](mailto:josegarcia_mex@hotmail.com)

**1. Similarity percentage between 16S rRNA gene sequences**

The 16S rRNA gene is ~1,500 nucleotide long and it is useful for microbiologists and microbial ecologists for various reasons. First, it is universally distributed among all Bacteria, which means that every single bacterial microorganism on Earth has at least one copy of this gene. Second, the 16S gene contains conserved groups of nucleotides that vary little among different types of Bacteria. We would not be able to align the sequences unambiguously if we would not have these conserved regions, thus hampering additional bioinformatics work. Finally, the 16S gene also contains variable and hypervariable regions, which allows us to catalogue Bacteria into groups based on differences in nucleotide composition. The evolution and classification of microbes, and, later on, the nucleotide composition and molecular evolutionary patterns of the 16S gene, have been the subject of intense research over the last decades.

As mentioned in the main text, the concept of Operational Taxonomic Unit (OTU) refers to groups of sequences that are more similar to each other compared to the rest. The similarity between any pair of nucleotide sequences can be expressed as a percentage, for instance two 1,500 nucleotide long sequences that are 100% similar have the exact same nucleotide composition. If, on the other hand, the sequences are only 10% similar, then they only share similarities in 150 nucleotides. Note that any similarity threshold is established regardless of the location of the differences or their position relative to each other (the differences can be located right to each other or spread throughout the entire length of the gene and this would still be considered the same).

Historically, a 97% similarity threshold was considered enough to cluster reference sequences into a particular OTU. A 97% similarity threshold involves about 45 nucleotides differences considering the full length of reference 16S gene sequences (~1,500 nucleotide long), or about 9 nucleotides per 300 nucleotides. A higher similarity threshold, say 99%, involves a lower difference in nucleotides, about 15 nucleotides throughout the entire length of the 16S gene, or 3 nucleotide difference per 300 nucleotides. If one considers a set of sequences, or any other things, the grouping of these things would yield more groups when considering a higher percentage of similarity. This is noticeable when looking at the differences in number of sequences between the reference OTU file from GreenGenes clustered at 97% similarity (99,322 sequences) and at 99% similarity (203,452 sequences). QIIME and others by default use a reference sequence file containing representative sequences clustered at 97% similarity but it is up to the researchers to use this reference file or others.

In the previous paragraph, we discussed sequence similarity percentage in a context of reference OTUs. Now, researchers often use (again) a 97% similarity in nucleotide composition to compare their unknown sequences against the reference sequences. However, during bioinformatics analysis this parameter can be changed at will. In QIIME, this is controlled in the similarity option of the pick_otus.py script. Interestingly, in this study the use of a higher percentage similarity (99%) to compare our unknown sequences with the reference OTUs showed lower numbers of OTUs in the closed approach (note that the reduction in the number of detected OTUs varied widely among the different studies) and more OTUs in the other approaches, using both the 97% (Supplemental Table S2) and the 99% (Supplemental Table S3) OTUs reference files.

| **Table S1. Summary of detected OTUs from the results obtained with 97% and 99% percentage similarity and the 97% OTU reference database.** | | | | | | |
| --- | --- | --- | --- | --- | --- | --- |
|  | **Closed** | | **De novo*** | | **Open** | |
| **Similarity** | **97%** | **99%** | **97%** | **99%** | **97%** | **99%** |
| Peach study | 758 | 440 | 1,549 | 3,183 | 1,603 | 2,758 |
| Wheat study | 1,302 | 15 | 37,474 | 95,586 | 8,686 | 15,743 |
| Quinoa study | 1,062 | 10 | 17,046 | 50,455 | 5,774 | 10,729 |
| Barley study | 1,078 | 8 | 15,599 | 46,309 | 5,366 | 10,095 |
| Cherry study | 2,439 | 388 | 138,203 | 736,873 | 69,658 | 213,425 |
| Raspberry study | 2,751 | 1,274 | 92,486 | 332,219 | 21,243 | 70,434 |
| Apple study | 2,095 | 152 | 153,681 | 579,600 | 69,010 | 153,877 |

*This approach does not consider any reference sequence database therefore the numbers are identical to the numbers in Table S3.

| **Table S2. Summary of detected OTUs from the results obtained with 97% and 99% percentage similarity and the 99% OTU reference database.** | | | | | | |
| --- | --- | --- | --- | --- | --- | --- |
|  | **Closed** | | **De novo*** | | **Open** | |
| **Similarity** | **97%** | **99%** | **97%** | **99%** | **97%** | **99%** |
| Peach study | 1,074 | 731 | 1,549 | 3,183 | 1,680 | 2,843 |
| Wheat study | 2,008 | 22 | 37,474 | 95,586 | 9,013 | 15,743 |
| Quinoa study | 1,606 | 14 | 17,046 | 50,455 | 5,976 | 10,755 |
| Barley study | 1,586 | 13 | 15,599 | 46,309 | 5,594 | 10,734 |
| Cherry study | 4,217 | 628 | 138,203 | 736,873 | 70,886 | 213,438 |
| Raspberry study | 4,433 | 2,247 | 92,486 | 332,219 | 21,834 | 71,850 |
| Apple study | 3,363 | 311 | 153,681 | 579,600 | 70,056 | 154,125 |

*This approach does not consider any reference sequence database therefore the numbers are identical to the numbers in Table S2.

**2. Information about diets**

The following Supplemental Table S1 contains all the information related to the diets used in the publications from which the data for this study came from.

**Table S3. Compositional information about all diets in the publications from which the data for this study came from.**

| **Publication** | **Animals, samples and experimental groups** | **Diets** |
| --- | --- | --- |
| **Peach (*Noratto et al. 2014*)** | **Male obese Zucker rats (Leprfa/Lepr+)** |  |
|  | Control obese (n=4) | Teklad Rodent Diet (300 kcal/100 g) |
|  | Obese Zucker rats with peach (n=4) | Teklad Rodent Diet (300 kcal/100 g) supplemented with peach juice ad libitum |
|  | Obese Zucker rats with plum (n=4) | Teklad Rodent Diet (300 kcal/100 g) supplemented with plum juice ad libitum |
| **Wheat (*Garcia-Mazcorro et al. 2016*)** | **Obese db/db and lean wild type male mice** |  |
|  | Control lean (n=11) | AIN-93 G Purified Rodent Diet (376 kcal/100 g) |
|  | Control obese (n=9) | AIN-93 G Purified Rodent Diet (376 kcal/100 g) |
|  | Obese with whole-wheat (n=10) | Diet based on 88% whole-wheat (387.76 kcal/100 g) |
| **Quinoa (*Garcia-Mazcorro, Mills & Noratto 2016*)** | **Obese db/db and lean wild type male mice** |  |
|  | Control lean (n=11) | AIN-93-G (376 kcal/100 g) |
|  | Control obese (n=10) | AIN-93-G (376 kcal/100 g) |
|  | Obese with quinoa (n=10) | Diet with 84% quinoa (377 kcal/100 g) |
| **Barley (*Garcia-Mazcorro et al. 2017*)** | **Obese db/db and lean wild type male mice** |  |
|  | Control lean (n=11) | AIN-93 G Purified Rodent Diet (376 kcal/100 g) |
|  | Control obese (n=10) | AIN-93 G Purified Rodent Diet (376 kcal/100 g) |
|  | Obese with barley (n=8) | Diet based on 88% barley (359 kcal/100 g) |
| **Cherry (*Garcia-Mazcorro et al. 2018*)** | **Obese db/db and lean wild type male mice** |  |
|  | Control lean (n=10) | AIN-93-G-MX Diet (198 kcal/100 g) |
|  | Control obese (n=10) | AIN-93-G-MX Diet (198 kcal/100 g) |
|  | Obese with cherry (n=12) | Modified AIN-93-G-MX Diet with 10% cherry powder (198 kcal/100 g) |
| **Raspberry (*Garcia-Mazcorro et al. 2018*)** | **Obese db/db male mice** |  |
|  | Control obese (n=15) | AIN-93G Diet (198 kcal/100 g) |
|  | Obese with raspberry (n=12) | Modified AIN-93G Diet with 5.3% raspberry supplementation |
| **Apple (*Garcia-Mazcorro et al. 2019*)** | **Dawley Sprague male rats** |  |
|  | Control high-fat (n=14) | Modified AIN-93G-MX (high-fat, 271 kcal/100 g, 60% from fat and 20% from carbohydrates) |
|  | High-fat with apple (n=14) | Modified AIN-93G-MX (high-fat with 5% freeze dried apple supplementation, 271 kcal/100 g, 60% from fat and 20% from carbohydrates) |
|  | Low-fat (n=5) | Modified AIN-93G-MX (low-fat, 271 kcal/100 g, 10% from fat and 70% from carbohydrates) |
|  | Low-fat with apple (n=6) | Modified AIN-93G-MX (low-fat with 5% freeze dried apple supplementation, 271 kcal/100 g, 10% from fat and 70% from carbohydrates) |

**3.** **UMAP**

We used uniform manifold approximation and projection (UMAP), a non-linear dimensionality reduction technique, to confirm the clusters that we observed using PCoA on unweighted UniFrac distances. The results confirmed the clustering of samples based on animal model and study (Figure S1).


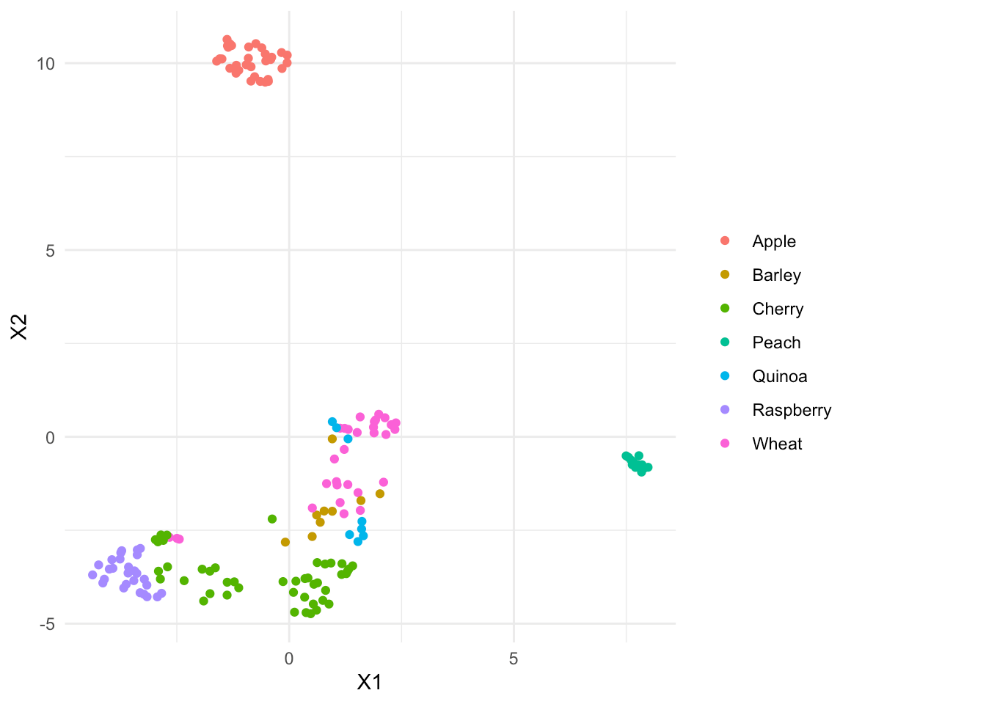


Figure S1. Plot showing UMAP results. The peach and apple studies were the only ones that used rats instead of mice.

**4. UniFrac analyses from closed97 approach on mice samples**

To discover any additional pattern or association between the microbial communities, we performed a separate analysis of mice samples only (n=120). Supplemental Figure S1 shows PCoA plots using unweighted UniFrac distances and Supplemental Table S4 summarize the results from the Adonis and ANOSIM tests of this additional analysis.


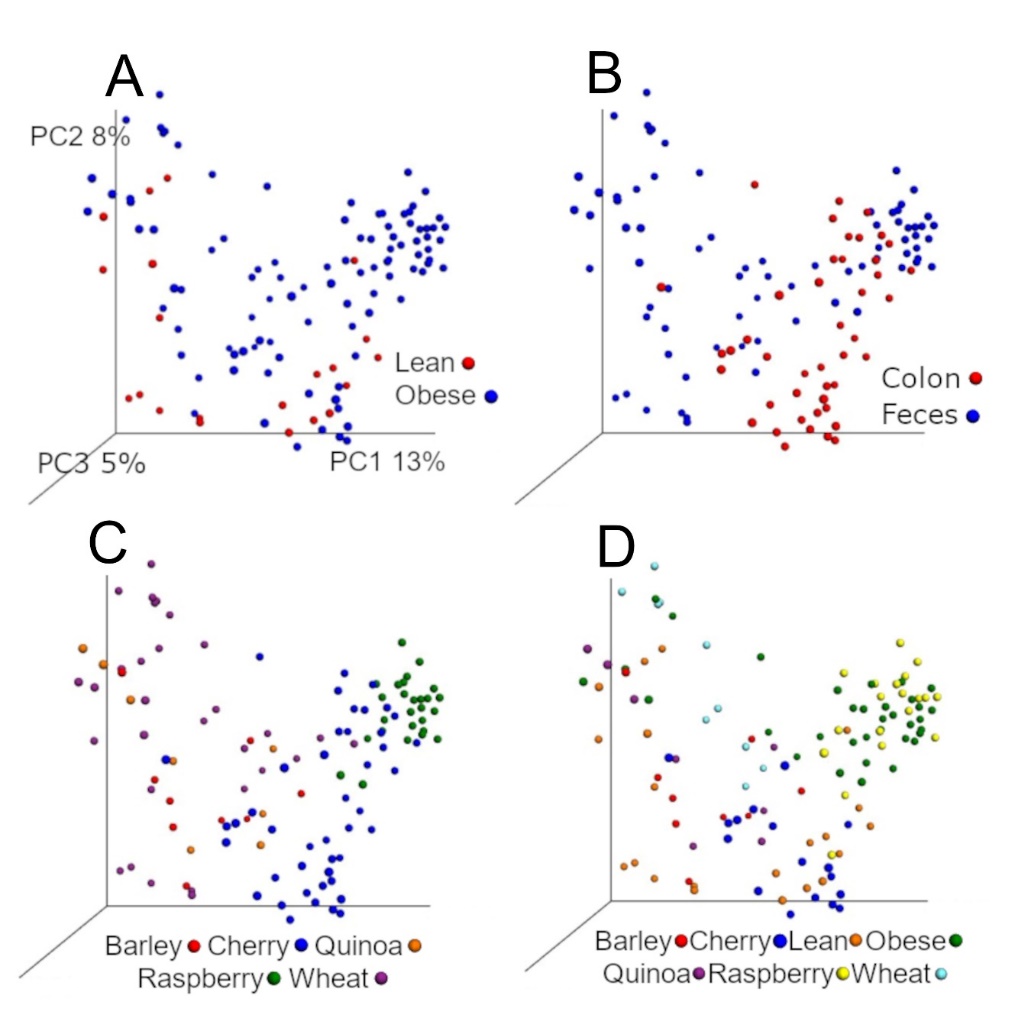


Figure S2. PCoA plots of unweighted UniFrac distances using data from the closed approach using the reference OTUs sequence file at 97% similarity (closed97 approach) with mice samples only (n=120). The plots highlight the effect of (A) obesity status, (B) anatomical site, (C) study, and (D) treatment. The values for each axis are only shown in A to facilitate viewing. These plots were built using a rarefaction depth of 100 sequences per sample to account for as many samples as possible (only two samples were left out using this rarefaction depth).

| **Table S4. Summary of results for mice samples (n=118) from the Adonis and ANOSIM tests for comparing categories using UniFrac data from the closed97 approach.** | | | | |
| --- | --- | --- | --- | --- |
|  | Adonis | | ANOSIM | |
|  | Unweighted | Weighted | Unweighted | Weighted |
| Treatment | *P* < 0.001  R^2^=19.8% | *P* < 0.001  R^2^=21.2% | *P* = 0.001  R=49.8% | *P* = 0.001  R=28.8% |
| Study | *P* < 0.001  R^2^=17.7% | *P* < 0.001  R^2^=11.4% | *P* = 0.001  R=48.9% | *P* = 0.001  R=22.9% |
| Obesity | *P* < 0.001  R^2^=4.2% | *P* < 0.001  R^2^=6.9% | *P* = 0.018  R=13.0% | *P* = 0.001  R=31.9% |
| Site | *P* < 0.001  R^2^=4.8% | *P* < 0.01  R^2^=2.4% | *P* = 0.004  R=10.3% | *P* = 0.850  R=-3.2 |

A rarefaction depth of 100 sequences per sample to account for as many samples as possible (only two samples were left out using this rarefaction depth). A total of 999 permutations were used to calculate the statistics.

**5. PICRUSt results**

PICRUSt is a tool that allows the prediction of functional profiles based on the nucleotide composition of the 16S gene. Supplemental Table S5 shows the most significant PICRUSt features for each of the factors investigated.

| **Table S5. Summary of the five PICRUSt features associated with the lowest *P* values for each of the factors investigated.** | | |
| --- | --- | --- |
| **Factor** | **Feature** | ***P* value** |
| Study | Ion channels | 5.7x10^-16^ |
|  | Ribosome Biogenesis | 1.6x10^-15^ |
|  | Phosphonate and phosphinate metabolism | 9.9x10^-15^ |
|  | Ribosome biogenesis in eukaryotes | 9.4x10-^14^ |
|  | Porphyrin and chlorophyll metabolism | 1.5x10^-13^ |
| Animal model | Ion channels | 0* |
|  | Tryptophan metabolism | 0* |
|  | Alpha-linoleic acid metabolism | 6.7x10^-14^ |
|  | Transcription machinery | 7.9x10^-13^ |
|  | Beta-alanine metabolism | 8.9x10^-13^ |
| Obesity status | *Vibrio cholera* pathogenic cycle | 1.2x10^-10^ |
|  | Bacterial toxins | 2.6x10^-5^ |
|  | Flavonoid biosynthesis | 3.2x10^-5^ |
|  | Alpha-linoleic acid metabolism | 5.2x10^-5^ |
|  | Fructose and mannose metabolism | 6.6x10^-5^ |
| Sequencing technique | Alpha-linoleic acid metabolism | 0* |
|  | RIG-I-like receptor signaling pathway | 0* |
|  | Aminoacyl-tRNA biosynthesis | 1.1x10^-18^ |
|  | Ascorbate and aldarate metabolism | 1.7x10^-14^ |
|  | Phosphotransferase system | 2.7x10^-14^ |
| Anatomical site | Cardiac muscle contraction | 8.2x10^-7^ |
|  | Small cell lung cancer | 5.1x10^-6^ |
|  | Viral myocarditis | 5.8x10^-6^ |
|  | Colorectal cancer | 6.6x10^-6^ |
|  | Parkinson’s disease | 7.1x10^-6^ |
| Treatment | Pentose phosphate pathway | 4.4x10^-16^ |
|  | Base excision repair | 9.9x10^-14^ |
|  | Flavonoid biosynthesis | 1.5x10^-13^ |
|  | DNA repair and recombination proteins | 1.8x10^-12^ |
|  | Flagellar assembly | 2.4x10^-12^ |

*P* values come from Welch’s t-test for factors with two levels (e.g. animal model), or ANOVA for factors with more than two levels. *P* values were adjusted using the Benjamini-Hochberg FDR test in STAMP. **P* values of 0 in STAMP are likely to be *P* values lower than 1x10^-18^. In this and other studies using PICRUSt, some features seem strange, such as cardiac muscle contraction or small cell lung cancer. Any inaccuracy in PICRUSt predictions is likely related to the lack of sequenced genomes from microbes related to the microbes found in the samples.

**6. BugBase results**

BugBase (https://bugbase.cs.umn.edu/index.html) is a tool that allows the prediction of phenotypes also based on the nucleotide composition of the 16S gene. Here you can find the results from BugBase from each study (Supplemental Figure S1 to Figure S7).


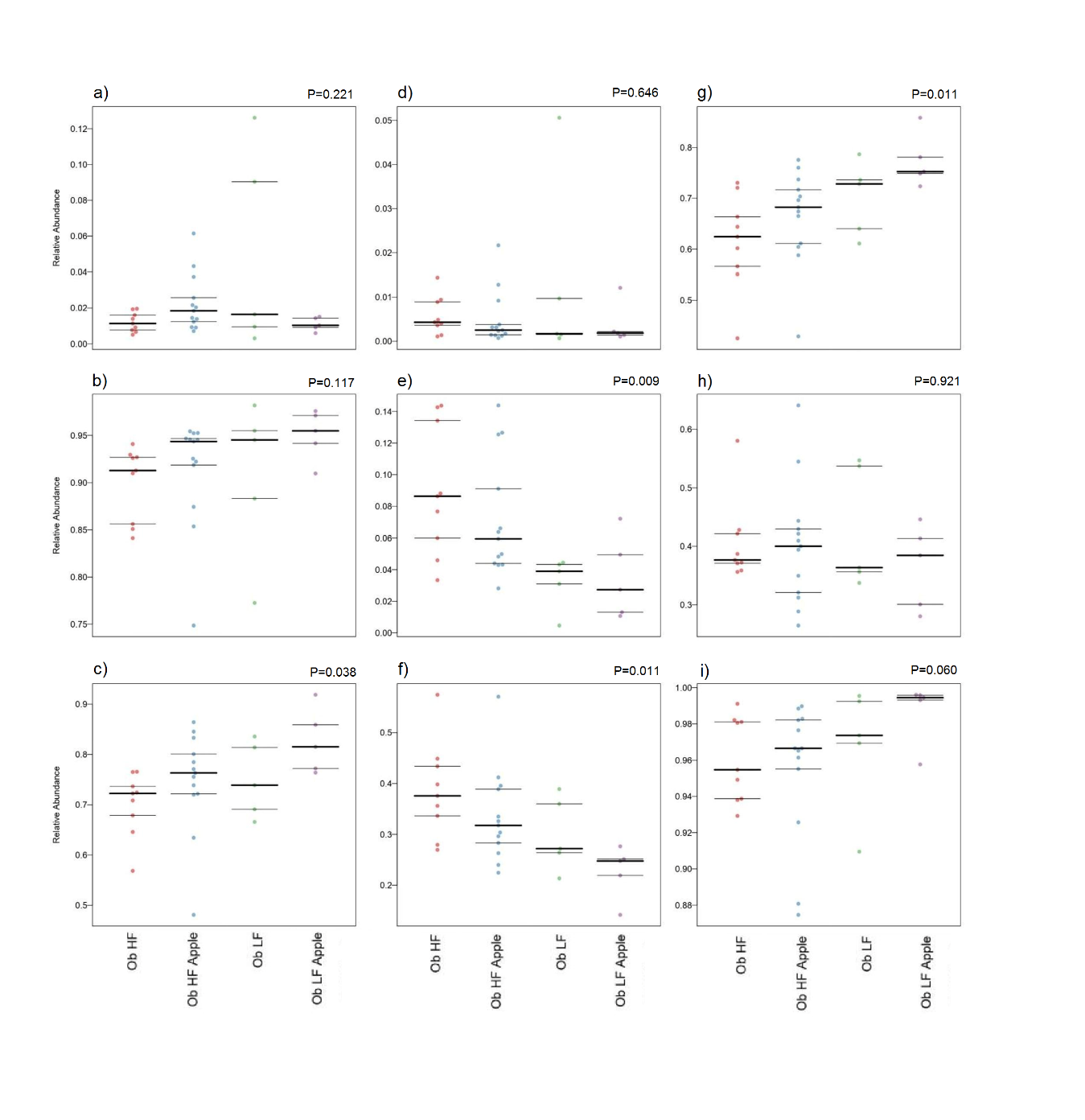


Figure S3. BugBase results for the apple study. a) aerobic Bacteria, b) anaerobic Bacteria, c) contains mobile elements, d) facultatively anaerobic, e) forms biofilms, f) gram negative, g) gram positive, h) potentially pathogenic, i) stress tolerant. The P value comes from the Kruskal-Wallis test performed by BugBase.


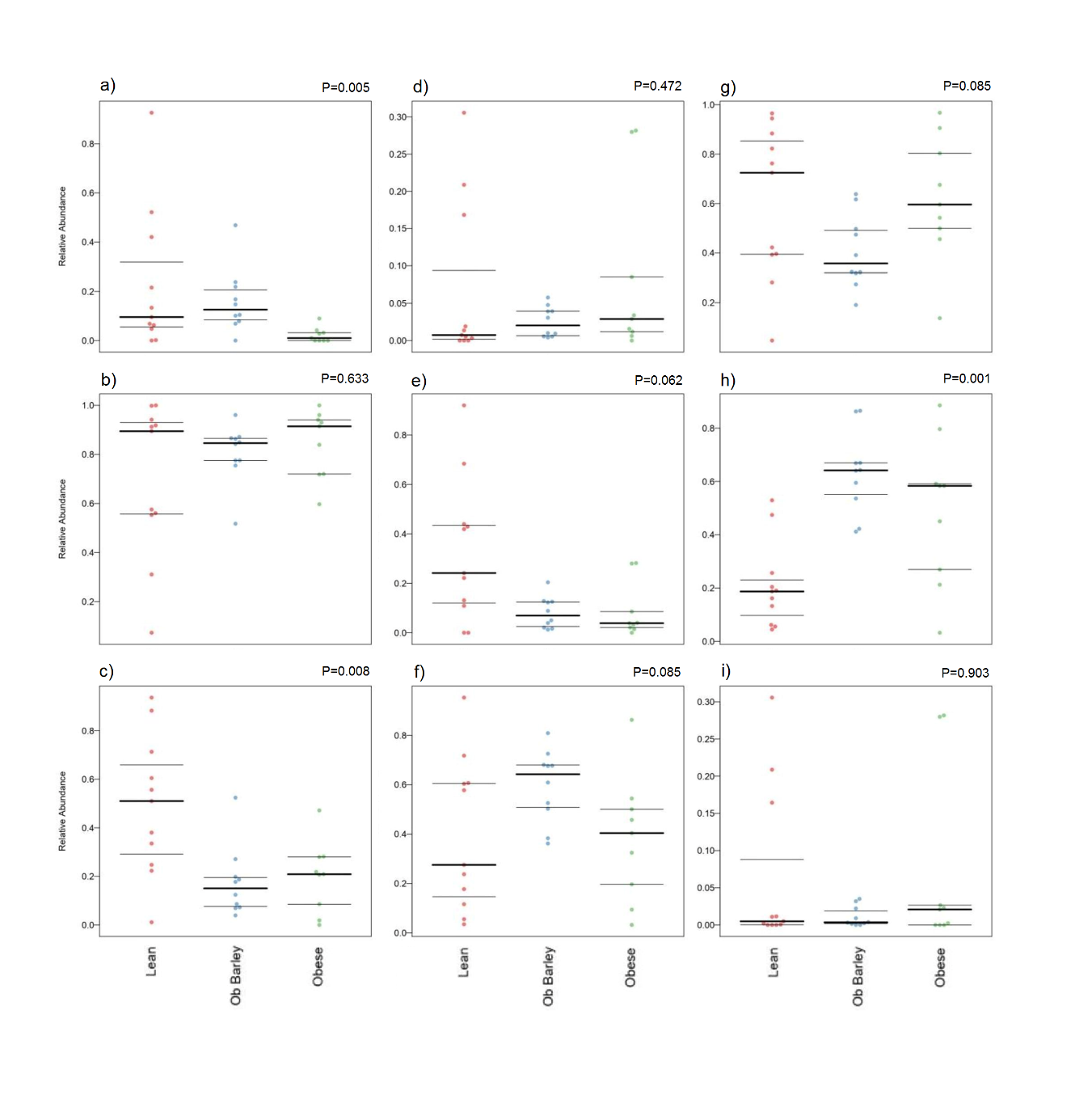


Figure S4. BugBase results for the barley study. a) aerobic Bacteria, b) anaerobic Bacteria, c) contains mobile elements, d) facultatively anaerobic, e) forms biofilms, f) gram negative, g) gram positive, h) potentially pathogenic, i) stress tolerant. The P value comes from the Kruskal-Wallis test performed by BugBase.


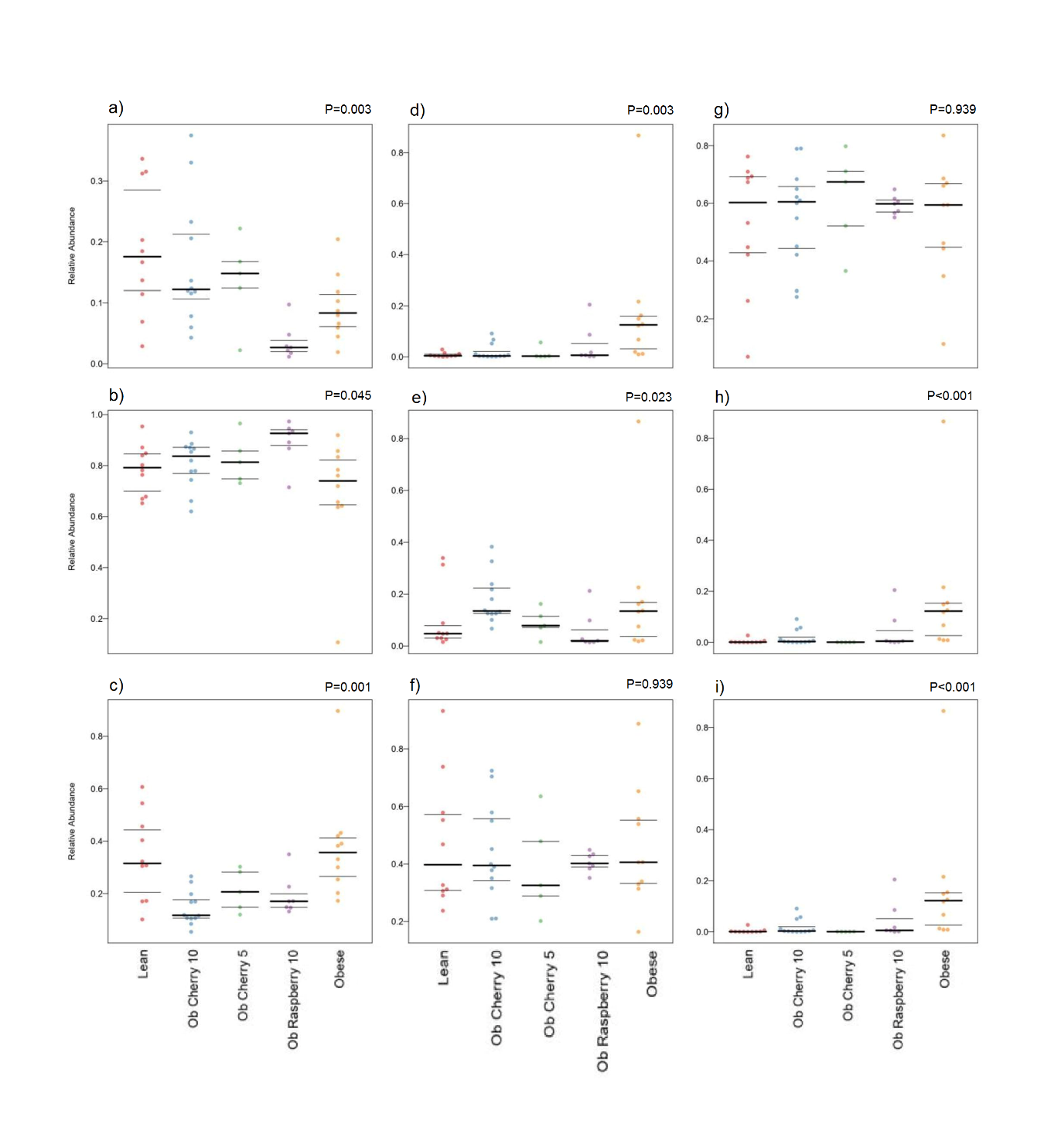


Figure S5. BugBase results for the cherry study. a) aerobic Bacteria, b) anaerobic Bacteria, c) contains mobile elements, d) facultatively anaerobic, e) forms biofilms, f) gram negative, g) gram positive, h) potentially pathogenic, i) stress tolerant. The P value comes from the Kruskal-Wallis test performed by BugBase.


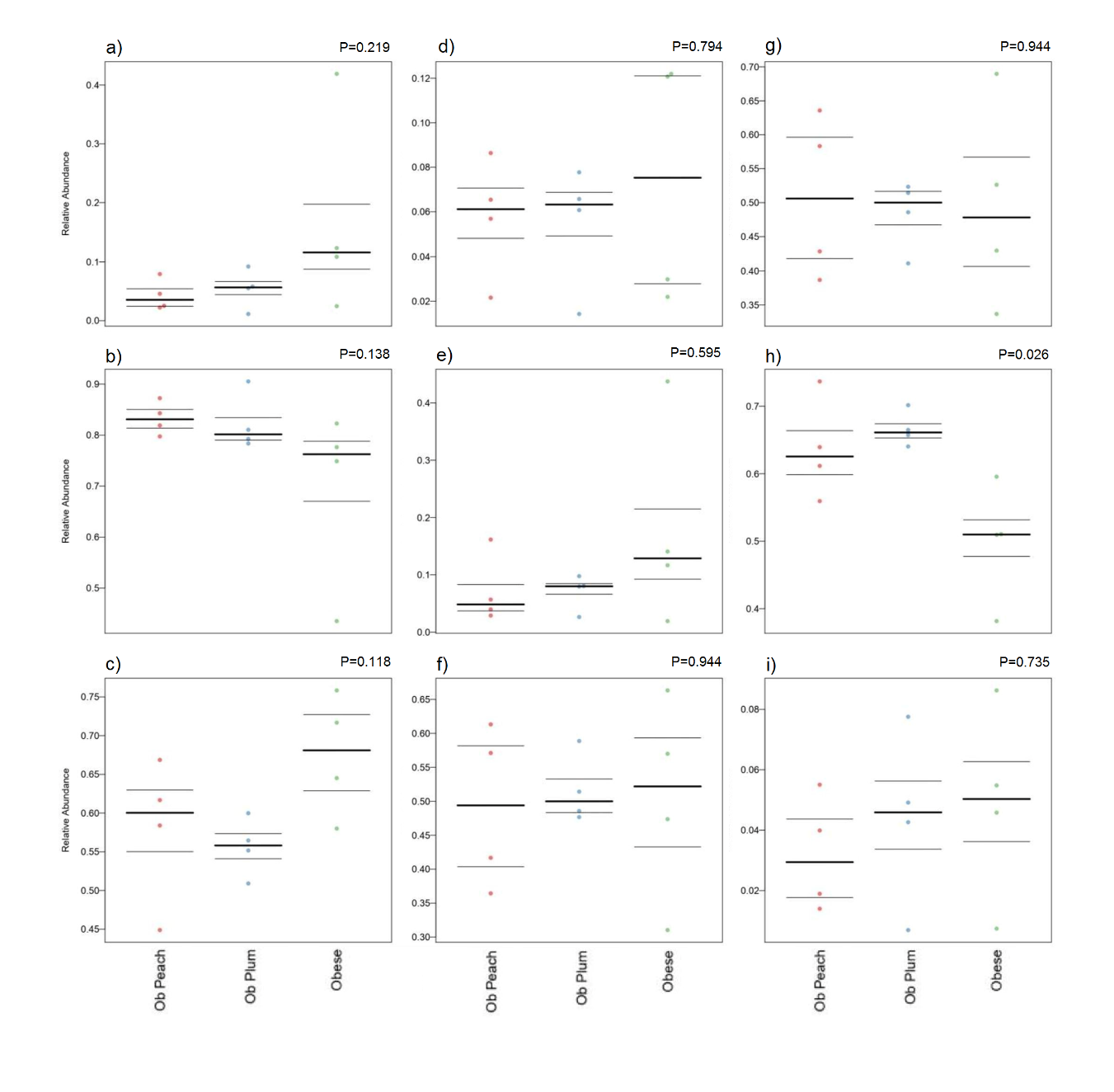


Figure S6. BugBase results for the peach study. a) aerobic Bacteria, b) anaerobic Bacteria, c) contains mobile elements, d) facultatively anaerobic, e) forms biofilms, f) gram negative, g) gram positive, h) potentially pathogenic, i) stress tolerant. The P value comes from the Kruskal-Wallis test performed by BugBase.


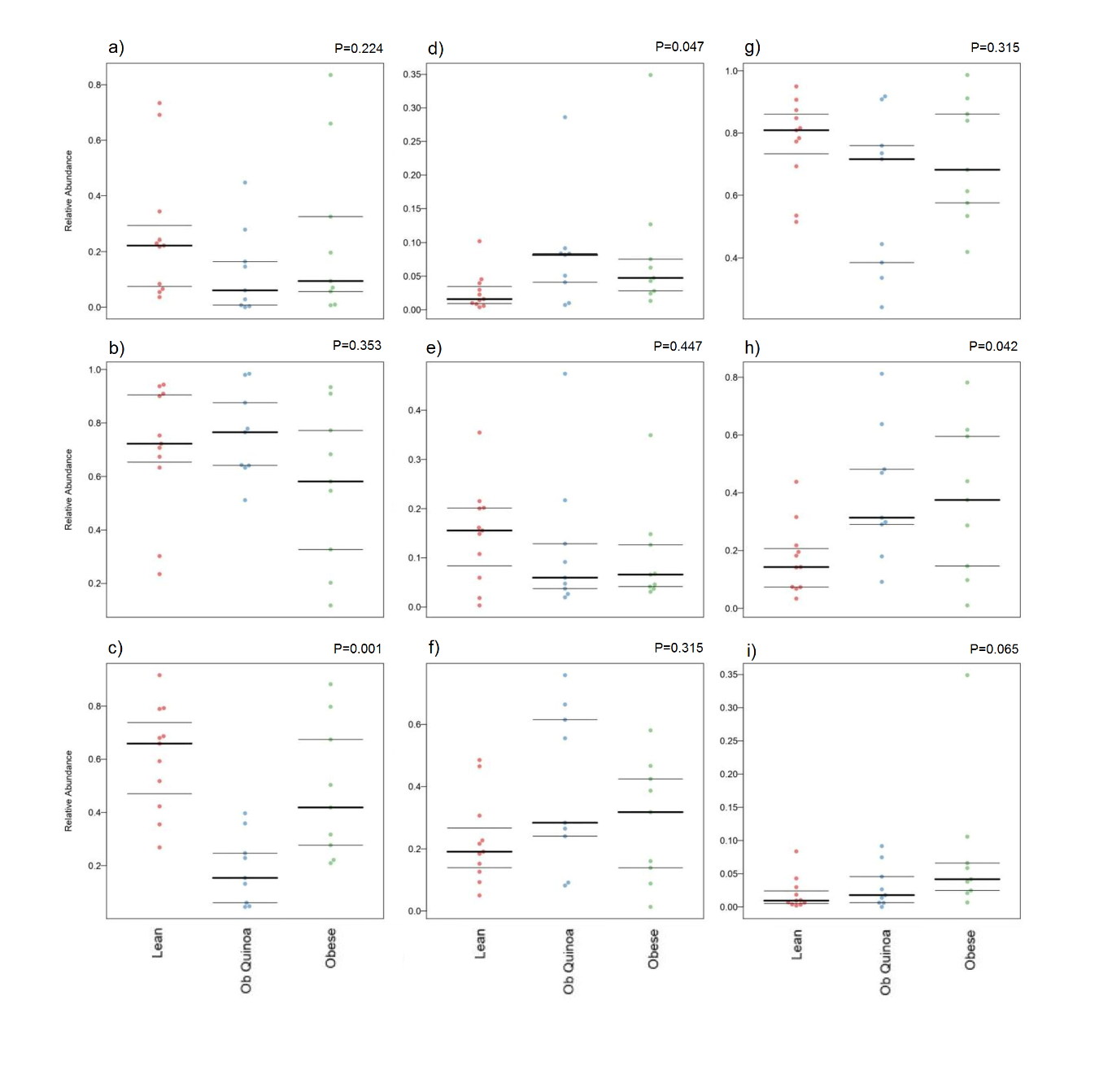


Figure S7. BugBase results for the quinoa study. a) aerobic Bacteria, b) anaerobic Bacteria, c) contains mobile elements, d) facultatively anaerobic, e) forms biofilms, f) gram negative, g) gram positive, h) potentially pathogenic, i) stress tolerant. The P value comes from the Kruskal-Wallis test performed by BugBase.


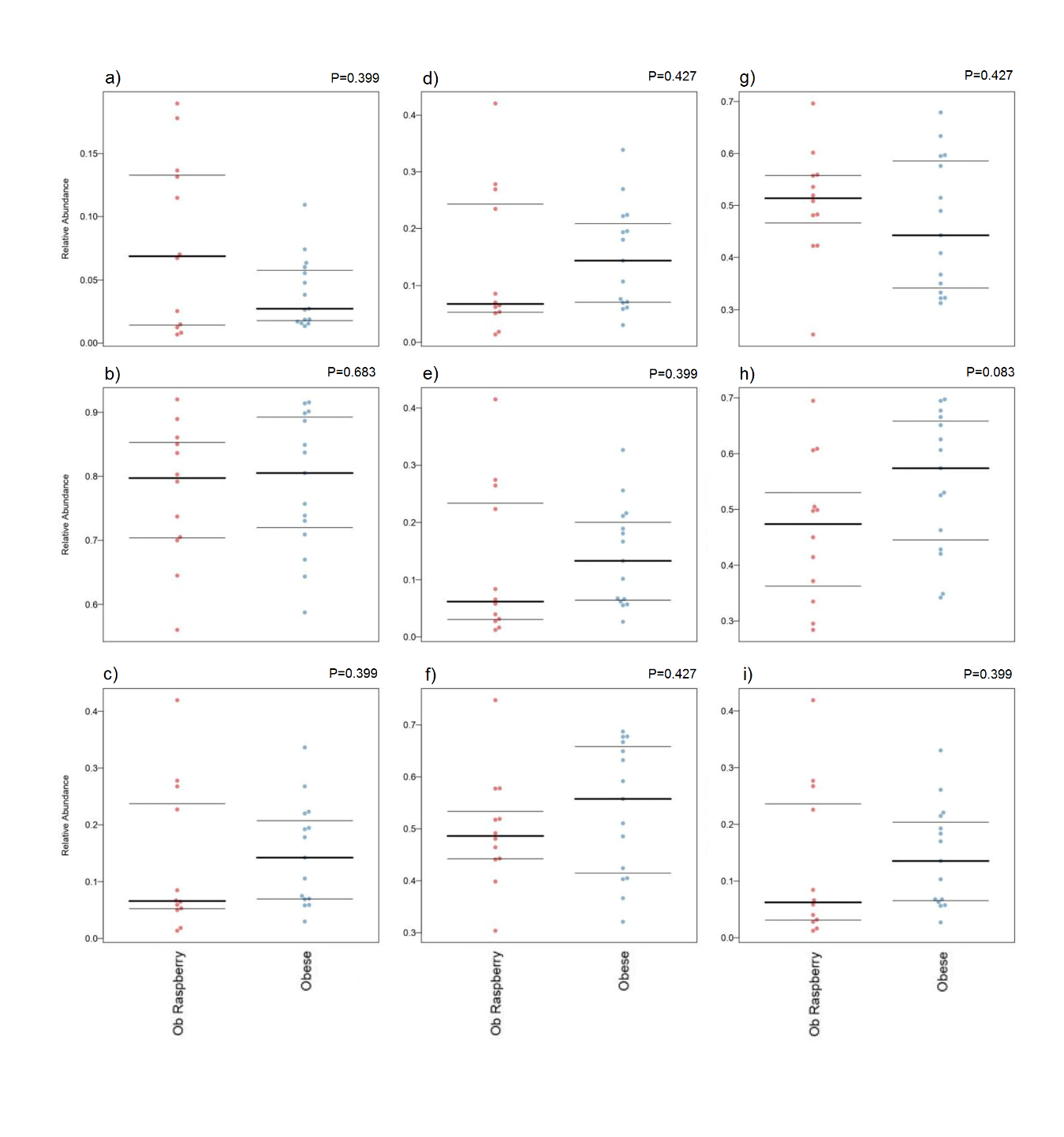


Figure S8. BugBase results for the raspberry study. a) aerobic Bacteria, b) anaerobic Bacteria, c) contains mobile elements, d) facultatively anaerobic, e) forms biofilms, f) gram negative, g) gram positive, h) potentially pathogenic, i) stress tolerant. The P value comes from the Mann-Whitney test performed by BugBase.


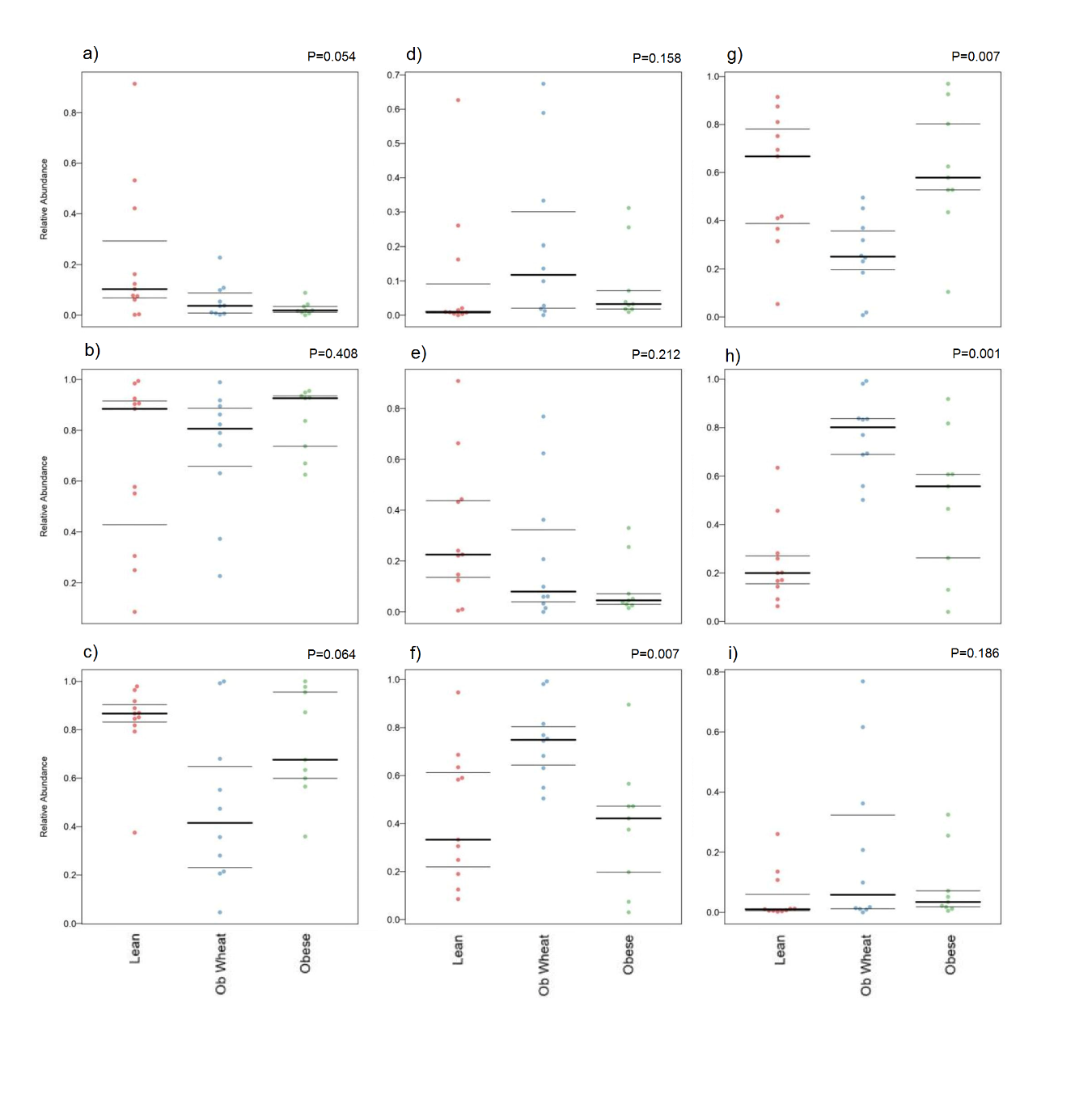


Figure S9. BugBase results for the wheat study. a) aerobic bacteria, b) anaerobic bacteria, c) contains mobile elements, d) facultatively anaerobic, e) forms biofilms, f) gram negative, g) gram positive, h) potentially pathogenic, i) stress tolerant. The P value comes from the Kruskal-Wallis test performed by BugBase.
